# Supplementary material for: Genome-Wide Identification and Expression Analysis of the Sucrose Synthase Gene Family in Sweet Potato and Its Two Diploid Relatives
Source: Int J Mol Sci. 2023 Aug 6;24(15):12493. doi: 10.3390/ijms241512493 (PMC10420203; doi:10.3390/ijms241512493)
Supplement: Supplementary file 1 [file ijms-24-12493-s001.zip › ijms-2500342-supplementary.pdf]

Table S1. Identification of *SUSs* in *I. batatas*, *I. trifida*, and *I. triloba*.

	Gene Name	Gene ID	Chr.	Gene Position
I. batatas	*IbSUS1*	*g505.t1*	LG1	2793890-2798579
	IbSUS2	*g5497.t1*	LG2	8562714-8568830
	IbSUS3	*g29617.t1*	LG7	31576704-31580891
	IbSUS4	*g30039.t1*	LG7	34437879-34441970
	IbSUS5	*g31210.t1*	LG8	5471709-5476663
	IbSUS6	*g55056.t1*	LG13	27660772-27665566
	IbSUS7	*g55074.t1*	LG13	27792067-27797658
	IbSUS8	*g55342.t1*	LG13	29268770-29272895
	IbSUS9	*g60893.t1*	LG15	6468996-6473927
I. trifida	*ItfSUS1*	*Itf05g23040.t1*	Chr05	23388562-23393210
	ItfSUS2	*Itf04g24160.t1*	Chr04	25686043-25692389
	ItfSUS3	*Itf03g05100.t1*	Chr03	3101581-3105775
	ItfSUS4	*Itf11g07860.t1*	Chr11	4351197-4356462
	ItfSUS5	*Itf02g04900.t1*	Chr02	6055942-6060284
	ItfSUS6	*Itf02g07130.t1*	Chr02	7203732-7209455
	ItfSUS7	*Itf06g18950.t1*	Chr06	21194480-21199532
I. triloba	*ItbSUS1*	*Itb05g23720.t1*	Chr05	28786401-28791478
	ItbSUS2	*Itb04g23580.t1*	Chr04	28590171-28596707
	ItbSUS3	*Itb03g05100.t1*	Chr03	3419915-3424462
	ItbSUS4	*Itb11g08290.t1*	Chr11	5423033-5428557
	ItbSUS5	*Itb02g00230.t1*	Chr02	198892-202920
	ItbSUS6	*Itb02g02420.t1*	Chr02	1386690-1392450
	ItbSUS7	*Itb06g17400.t1*	Chr06	21252081-21257133

Table S2. Primers used in this study.

Gene	Forward Primer	Reverse Primer
actin	AGCAGCATGAAGATTAAGGTTGTAGCAC	TGGAAAATTAGAAGCACTTCCTGTGAAC
IbSUS1	AACAAAATGCGACCAGGAAC	ACCAAATTCCCATCGGTGTA
IbSUS2	AGGTGTTTGGGAGTTTGTGC	ACCCCATTTCCAATTGATGA
IbSUS3	TCAATCTGGTTGTGGTTGGA	GCGGTAGAGTTCGCTGTTTC
IbSUS4	GTGGTTGGAGGTTCCTTTGA	GCGGTAGAGTTCGCTGTTTC
IbSUS5	GAACAGGCTGCTGAGCTCTT	CGCCATAACTTGCGGTATTT
IbSUS6	AACTAGGTTGTTGCCGGATG	GCAACATCCTCGGTGAAAGT
IbSUS7	CCTGCCAATGGTAACTTCGT	CGTCTTGCCCTTGTATTGGT
IbSUS8	TTCTTGCCAATTCACAGCAG	TGCCTGAAACAACTCTGCAC
IbSUS9	GCCAACCCTTACCAAGTCAA	GGACGTTTTGGAGTGTCGTT

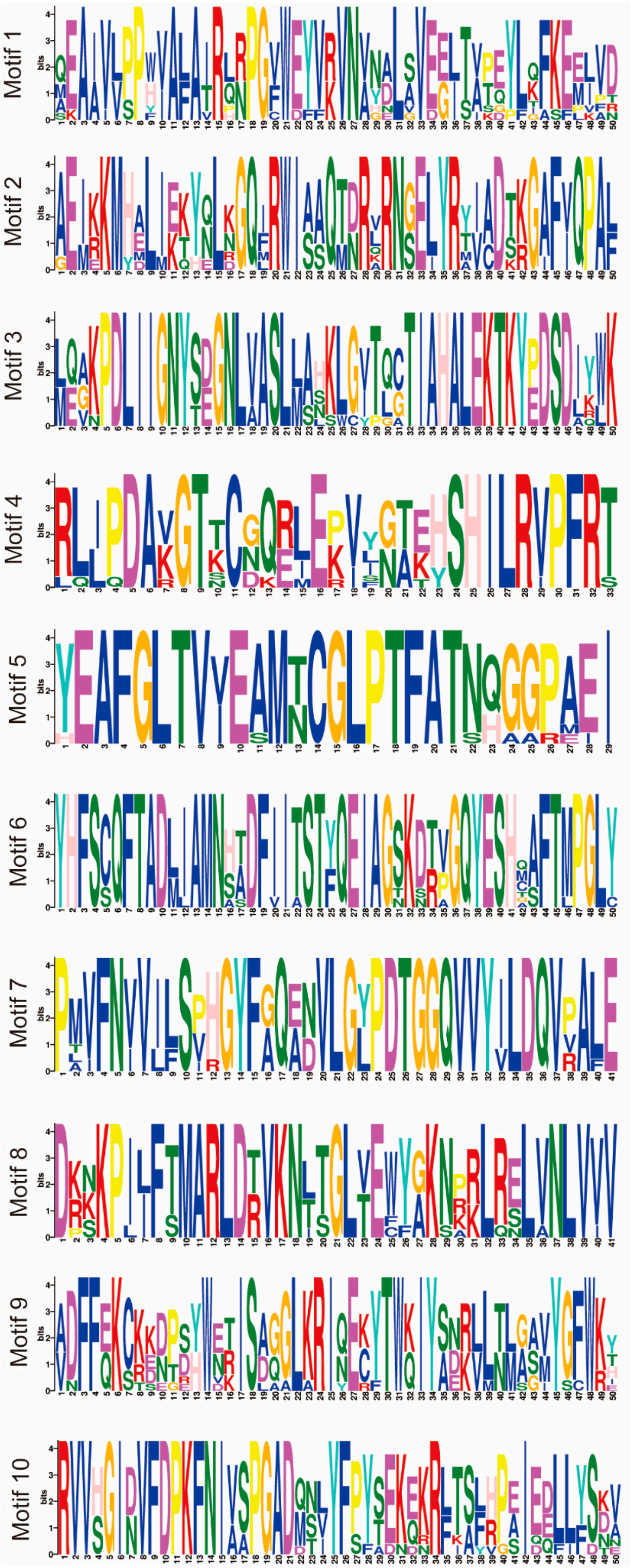

Figure S1. Sequence logos of the 10 conserved motifs.

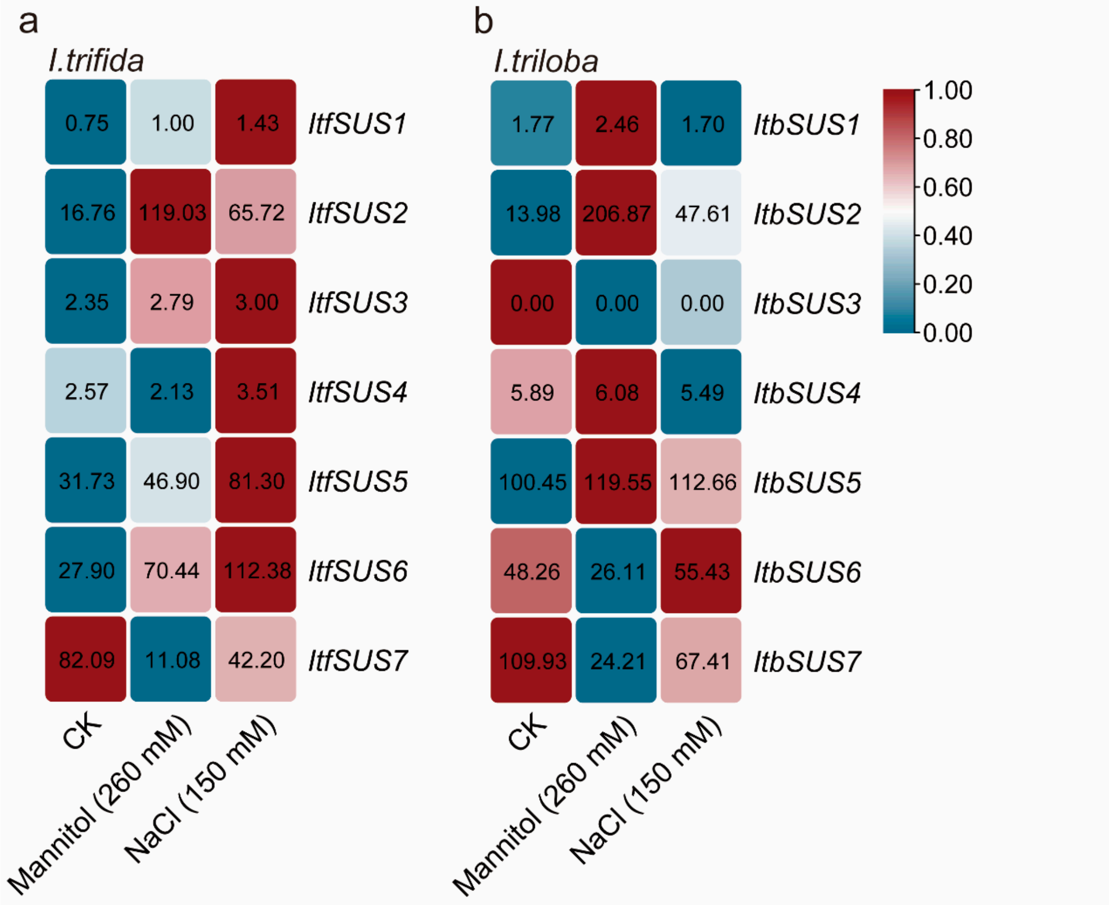

Figure S2. Expression analysis of *ItfSUSs* (**a**) and *ItbSUSs* (**b**) in response to drought and salt stresses as determined by RNA-seq. FPKM values are shown in the boxes.

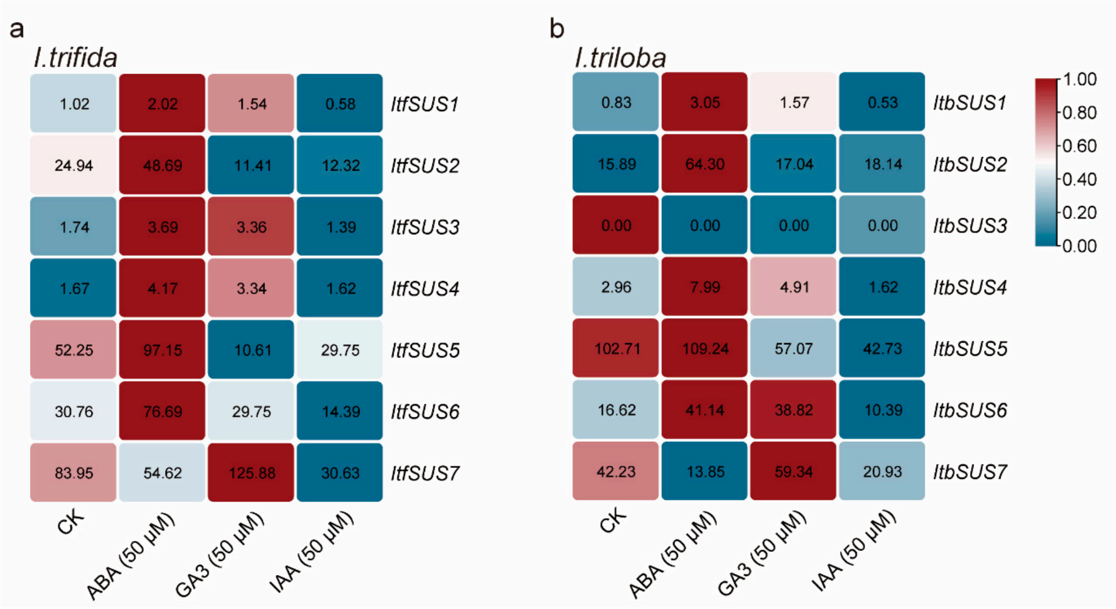

Figure S3. Expression analysis of *ItfSUSs* (**a**) and *ItbSUSs* (**b**) in response to different hormones (ABA, GA3, and IAA) as determined by RNA-seq. FPKM values are shown in the boxes.
